# Supplementary material for: Rotating neurons for all-analog implementation of cyclic reservoir computing
Source: Nat Commun. 2022 Mar 23;13:1549. doi: 10.1038/s41467-022-29260-1 (PMC8943160; doi:10.1038/s41467-022-29260-1)
Supplement: Supplementary file 1 — Supplementary Information [file 41467_2022_29260_MOESM1_ESM.pdf]

Supplementary Information

Rotating neurons for all-analog implementation of cyclic reservoir computing

Xiangpeng Liang[1,2,†], Yanan Zhong[1,3,†], Jianshi Tang[1,4*], Zhengwu Liu[1], Peng Yao[1], Keyang Sun[1], Qingtian Zhang[1,4], Bin Gao[1,4], Hadi Heidari[2*], He Qian[1,4], Huaqiang Wu[1,4*]

[1]School of Integrated Circuits, Beijing National Research Center for Information Science and Technology (BNRist), Tsinghua University, Beijing, 100084, China

[2]Microelectronics Lab, James Watt School of Engineering, University of Glasgow, Glasgow, G12 8QQ, United Kingdom

[3]Institute of Functional Nano & Soft Materials (FUNSOM), Jiangsu Key Laboratory for Carbon-Based Functional Materials & Devices, Soochow University, Suzhou, Jiangsu, 215123, China

[4]Beijing Innovation Center for Future Chips (ICFC), Tsinghua University, Beijing, 100084, China

[*]Email: jtang@tsinghua.edu.cn, hadi.heidari@glasgow.ac.uk, wuhq@tsinghua.edu.cn.

[†]These authors contributed equally: Xiangpeng Liang and Yanan Zhong

This **Supplementary Information** includes:

Figure S1. Experimental results for Mackey-Glass chaotic signal with $\tau > 17$

Figure S2. Memristor-based fully connected output layer.

Figure S3. Normalized output weights and error.

Figure S4. Handwriting recognition result for each participant.

Table S1. Comparison with literature-reported reservoir systems.

Table S2. Power breakdown for 8 × 8 eRNR system (μW).

Note S1. Reservoir characteristics.

Note S2. Demonstration and data acquisition platform.

Note S3. Why eRNR can be more resource-efficient?

Supplementary Figure

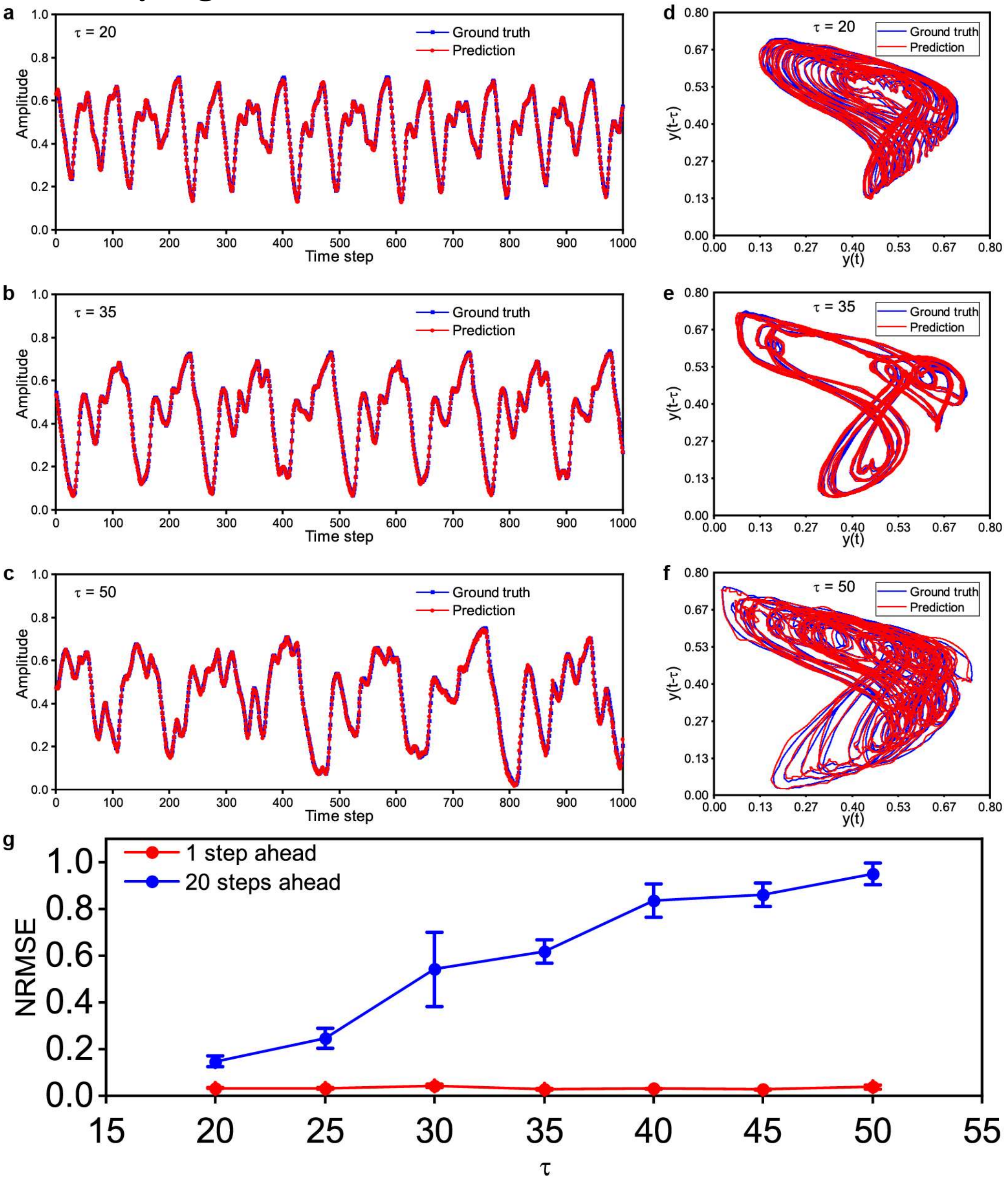

Supplementary Figure 1. Experimental results for Mackey-Glass chaotic signal prediction with $\tau > 17$. a, b, c, Three episodes of one-step ahead prediction of Mackey-Glass time series result compared with the ground truth using the chaotic signal with τ = (**a**) 20, (**b**) 35 and (**c**) 50. **d, e, f,** Phase space of the prediction compared with ground truth using the chaotic signal with τ = (**d**) 20, (**e**) 35 and (**f**) 50. **g,** NRMSE results of 1 and 20 steps ahead prediction with varied τ values.

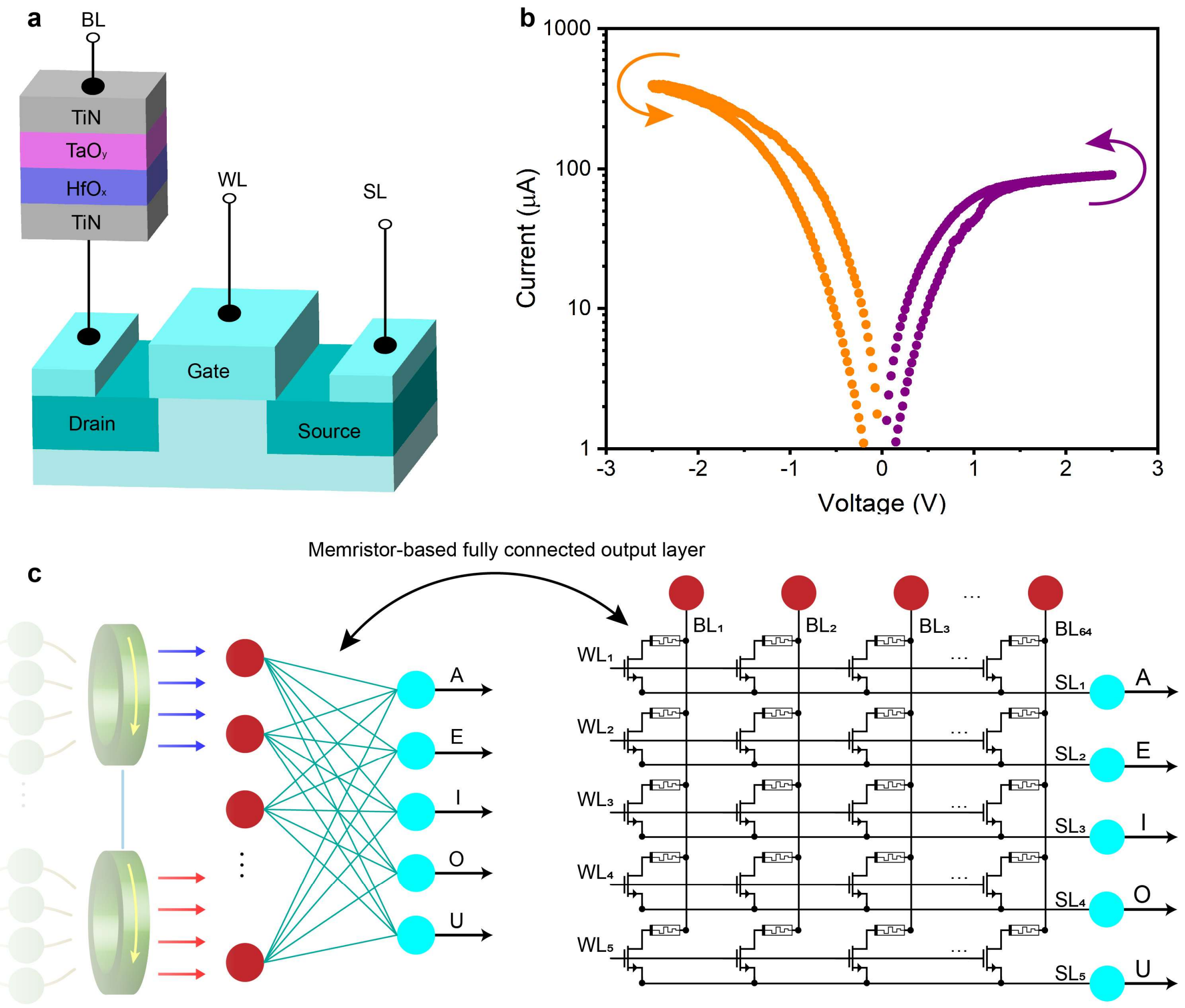

Supplementary Figure 2. Memristor-based fully connected output layer. a, Schematic of the 1T1R cell consisting of one transistor and one TiN/HfO_x/TaO_y/TiN memristor. **b**, DC I-V characteristics of the memristor. **c**, memristor-based fully connected output layer implemented by the 1T1R array. The WL, BL and SL indicate the word line, bit line and source line, respectively.

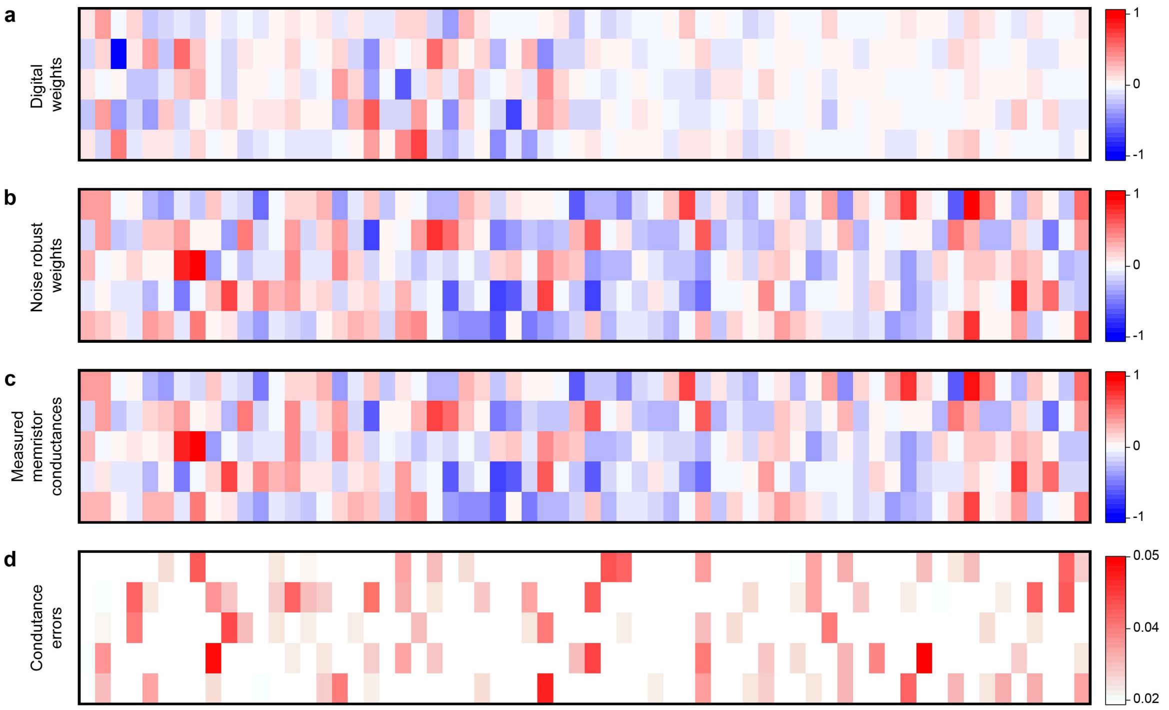

Supplementary Figure 3. Normalized output weights and error. a, Output weights without noise-aware training. **b,** Output weights with noise-aware training. **c,** Analog output weights measured from the memristor array. **d,** weights error resulted from the difference between the measured and target memristor conductance.

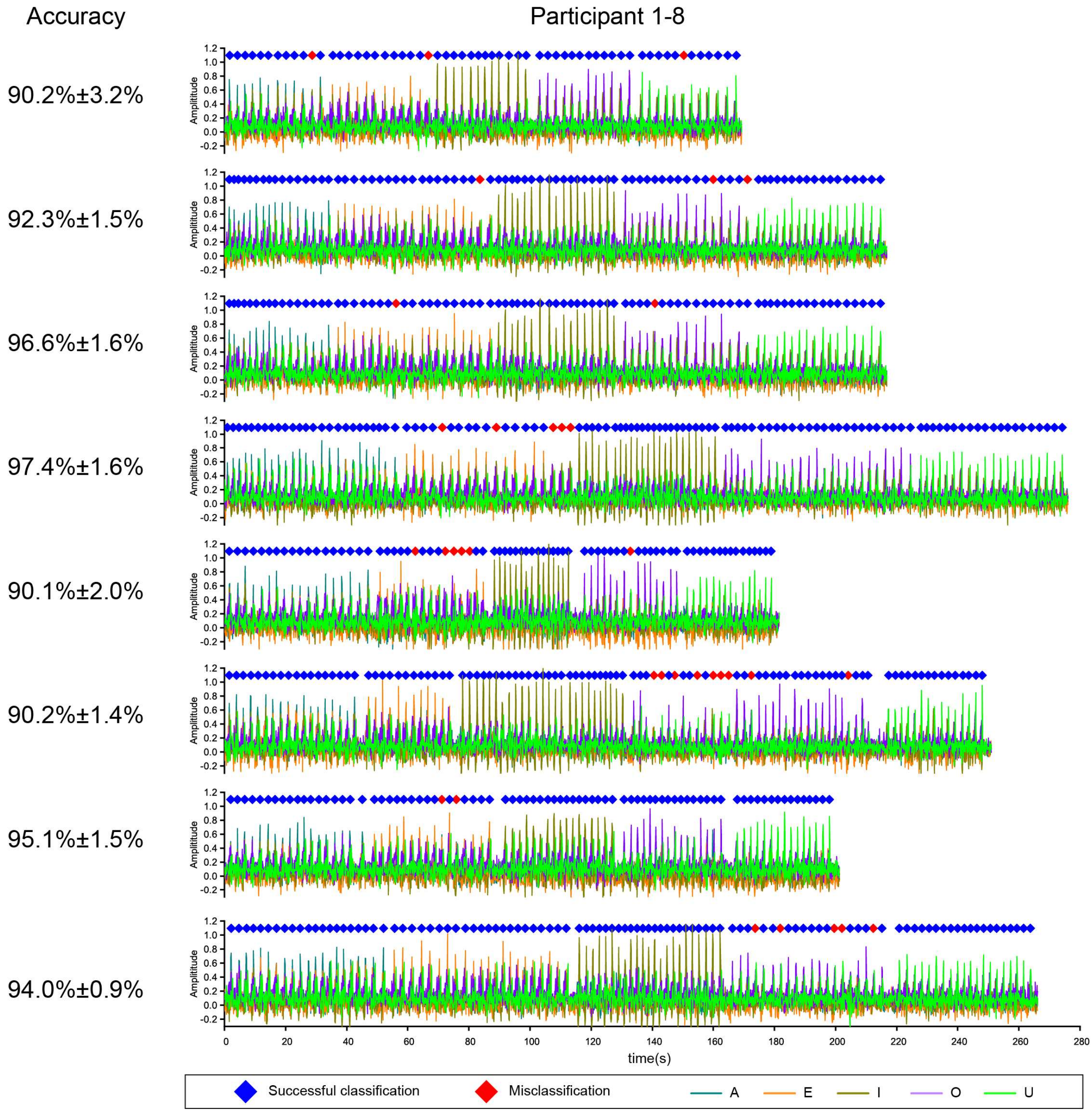

Supplementary Figure 4. Handwriting recognition result for each participant.

Supplementary Table 1. Comparison with system-level power of literature-reported reservoir systems

Reference	Implementation	*N*	Processing rate (Hz)	Power
Alomar et. al.[1]	FPGA	48	10^6	1.5W
Kleyko et. al.[2]	FPGA	100	-	1.6W
Alomar et. al.[3]	FPGA	50	1142	83mW
Brunner et. al.[4]	Optoelectronic	388	13×10^6	150W
This work	All-analog eRNR	64	10	32.7μW
			1×10^3	32.9μW
			100×10^3	79.0μW
			10×10^6	4.7mW

Supplementary Table 2. Simulated power breakdown for 8 × 8 eRNR system (μW)

Processing rate (Hz)	eRNR			Memristor	Total power (μW)
	Counter	Rotor	Neurons		
10	0.93	5.59	26.16	46.3×10^{-4}	32.7
1×10^{3}	0.93	5.59		46.3×10^{-2}	32.9
100×10^{3}	0.96	5.64		46.3	79.0
10×10^{6}	3.98	11.03		4633.6	4674.8

Supplementary Note 1. Reservoir characteristics

In this article, four task-independent network characteristics, memory capacity (MC), kernel quality (KQ), generalization rank (GR) and computing ability (CA) are used to compare different reservoirs[5].

1) Memory capacity (MC)

MC is frequently used to evaluate a reservoir's capability to retain history information within the fading memory. The MC can be quantified by a binary sequence reconstruction task. In this task, a binary sequence is randomly generated: u(k)=1 or -1, and $x_i(k)$ is the shifted version of u(k) by *i* steps: $x_i(k)=u(k-i)$, for i=1, 2, …∞. In our simulation, 4000 data points of u(k) were generated, in which 3000 points were used in training and 1000 points were used in testing. The u(k) was injected into the reservoir, resulting in the state output **s(k)**. The training state matrix was used to calculated $\mathbf{W}_{\mathbf{out}}$ using Ridge regression and the target was $x_i(k)$, for k∈[1,3000]. In the testing phase, the testing state matrix (**s(k)** for k∈[3001, 4000]) was multiplied by the trained $\mathbf{W}_{\mathbf{out}}$ to obtain $y_i(k)$, which indicated the reconstruction result. If the resulting $y_i(k)$ can properly match the testing $x_i(k)$, the reservoir can retain the information i steps ahead, which implies its memory property. Then, the MC can be quantified by taking the sum of the linear correlation between reconstruction $y_i(k)$ and actual shifted sequence $x_i(k)$ for *i* going from 1 to infinity:

$$MC = \sum_{i=1}^{\infty} \rho(y_i(k), x_i(k)) = \sum_{i=1}^{\infty} \frac{< y_i(k), x_i(k) >_k^2}{\sigma^2(u(k))\sigma^2(x_i(k))} \quad (1)$$

where ρ denotes the correlation and *s* is the standard deviation. The sequence u(k) was randomly generated and kept unchanged throughout the simulation to make the MC of different reservoirs and parameters comparable.

2) Kernel quality (KQ)

KQ evaluates the reservoir's performance on a high-dimensional nonlinear mapping. An ideal reservoir should be able to generate linearly separable node states in a higher dimensional

space for different inputs. For an N-neuron reservoir, we first generate N different input sequences U=[u_1, u_2…u_N] and every u_i contains k random values. The N sequences will be injected into the reservoir one by one. For every sequence, the state matrix generated by k^{th} data is of interest, while the first k-1 data are employed to initialize the reservoir dynamic. Collecting all k^{th} state matrix forms an $N \times N$ matrix. Ideally, the states generated by different input sequences should be linearly independent, which means the rank of the $N \times N$ matrix should be equal to N. Therefore, this normalized rank can measure the high-dimensional mapping quality, also known as KQ.

3) Generalization rank (GR)

GR tests the reservoir's response for similar inputs. The reservoir should react to the coming temporal and deliver their representative states onto their classes regardless the effect of the previous inputs and minor fluctuation. Otherwise, the reservoir would collapse into an unwanted chaotic system that is highly sensitive to the initial condition and noise. To test the GR, a random sequence u_x with a length of l was generated to connect to the end of every u_i used in the KQ to form a new sequence U' = [[u_1 u_x], [u_2 u_x]…[u_N u_x]]. It means that, for every reservoir under tested, it is first fed by different sequence with random values. Followed by all random values, the same sequence u_x with length l is fed into all reservoirs. Again, the states collected at the end or $(k+l)^{th}$ input form the N×N matrix to calculate the rank with 0.01 tolerance. In contrast to the KQ, a low rank means that the reservoir can quickly shift its attention to the latest l inputs, which is more desirable. Note that the choice of l should be determined by the temporal signal in actual application. For example, in the NARMA10 task, we understand that each point of NARMA10 is highly relevant to the previous 10 points. Therefore, a reservoir that gains a low GR when l = 10 can better couple with the NARMA10 system for prediction.

4) Computing ability (CA)

CA is simply a combination of KQ and GR. It has been defined that a reservoir with good CA should be able to quickly focus on the certain length of previous inputs (low GR) and map it onto a linearly separable space (high KQ). Thus, CA can be calculated by the difference between them: CA = KQ - GR.

Supplementary Note 2. Demonstration and data acquisition platform

The schematic of eRNR is shown in Fig. 2. The network size of our prototype is $N = 8$ and $M = 8$, which means the single eRNR consists of 8 neurons and there are 8 parallel eRNRs. Both pre- and post-neuron rotors were implemented by eight CD4051B which is an 8-channel analog multiplexer from Texas Instrument. The three signal selection ports were connected to a 3-bit binary counter consisted of a 4-bit counter (74LS161) and an inverter (74HC04). The input mask was implemented by 8 switches to select positive or negative signals. In order to improve the state richness, each eRNR circuit should use a different input mask configuration. The customized demonstration and data acquisition platform serves to interface with the eRNR hardware, collect experimental data, and perform real-time demonstrations but regardless of low-power design. The 64 state channels on the eRNRs were connected to 8 12-bit ADC channels on a STM32 microcontroller via 8 multiplexers. There are additional 2 ADC channels on STM32 for collecting sensory signals. The total 66 channels data were sent to PC via universal asynchronous receiver/transmitter (UART) communication. The user interface software developed on LabVIEW received the data packages and plotted them in real-time. The software can also store the data in a file for further processing.

In the real-time Mackey-Glass signal prediction demonstration, the software can also execute a MATLAB script where the Ridge regression was performed to calculate the $\mathbf{W}_{\mathbf{out}}$. Then, the $\mathbf{W}_{\mathbf{out}}$ was multiplied by the 64 state values at every time step to output the prediction result that was plotted in both the time-amplitude window and the phase window. In the handwriting recognition task, the platform collected the handwriting and state data for every participant. The $\mathbf{W}_{\mathbf{out}}$ was trained in MATLAB by post-processing all the data from 8 participants. After training, the software can read the trained weights and calculate the 5 channels result indicating the probabilities of the 5 vowel classes. Both demonstrations mentioned above can be found in the **Supplementary Video 1** and **2**.

Supplementary Note 3. Why eRNR can be more resource-efficient?

From a fundamental perspective, the different mechanisms of introducing memory in rotation-based architecture and other architectures mainly determine their power efficiency. In the rotation-based architecture, the memory is provided by the rotating dynamic node itself (see **Fig. 3a** and **Methods**). The excellent consistency between the rotation behavior and software algorithm frees the system from using extra control units, ADC, buffer and memory, which remarkably reduce the system complexity and power consumption. Also, implementing the logic switches for rotation is a resource-efficient use of CMOS-based transmission gates. Meanwhile, the rotating dynamic node serves to process signal and retain previous information simultaneously. Such in-memory computing paradigm is advantageous for low-power computing. In other architectures, such as the well-studied delay-based approach, the memory is actually separated from the processor. Although carrying out the processing in the nonlinear dynamic node was a significant progress, the memory is mainly provided by the delay unit which is constrained by the limitations of conventional digital computing, such as power consumption, throughput and latency[6]. These fundamental differences result in the better power efficiency for the proposed rotation-based architecture.

Compared with the classic random reservoir computing, the key difference of cyclic reservoir is the connection in the reservoir layer defined by $\mathbf{W_{res}}$. The $\mathbf{W_{res}}$ of random reservoir is a randomly generated matrix with a proper spectral radius, while the cyclic counterpart is a shifted identity matrix which can be implemented in a more deterministic manner without performance degradation[7]. In this work, it has been proven that the cyclic $\mathbf{W_{res}}$ can be equivalent to a physical rotor (see **Methods**), while an effective physical counterpart of random $\mathbf{W_{res}}$ is yet to be found, which remains an exciting challenge to be addressed for future studies.

Supplementary References

1 Alomar, M. L. *et al.* Efficient parallel implementation of reservoir computing systems. *Neural Computing and Applications* **32**, 2299-2313, (2020).

2 Kleyko, D., Frady, E. P., Kheffache, M. & Osipov, E. Integer Echo State Networks: Efficient Reservoir Computing for Digital Hardware. *IEEE Trans. Neural Networks Learn. Syst.*, 1-14, (2020).

3 Alomar, M. L. *et al.* Digital Implementation of a Single Dynamical Node Reservoir Computer. *IEEE Trans. Circuits Syst. II Express Briefs* **62**, 977-981, (2015).

4 Brunner, D., Soriano, M. C., Mirasso, C. R. & Fischer, I. Parallel photonic information processing at gigabyte per second data rates using transient states. *Nat. Commun.* **4**, 1-7, (2013).

5 Appeltant, L. *Reservoir computing based on delay-dynamical systems* Doctoral thesis, (2012).

6 Indiveri, G. & Liu, S. Memory and Information Processing in Neuromorphic Systems. *Proc. IEEE* **103**, 1379-1397, (2015).

7 Rodan, A. & Tino, P. Minimum Complexity Echo State Network. *IEEE Trans. Neural Networks* **22**, 131-144, (2011).
