# Supplementary material for: Rotating neurons for all-analog implementation of cyclic reservoir computing
Source: Nat Commun. 2022 Mar 23;13:1549. doi: 10.1038/s41467-022-29260-1 (PMC8943160; doi:10.1038/s41467-022-29260-1)
Supplement: Supplementary file 3 — Description of additional Supplementary File [file 41467_2022_29260_MOESM3_ESM.pdf]

Descriptions of Additional Supplementary Information files

Supplementary Movie 1. Real-time Mackey Glass time series prediction

Supplementary Movie 2. Near-sensor reservoir computing for handwritten recognition
